# Integrating network pharmacology and experimental validation to explore the effect and mechanism of AD-1 in the treatment of colorectal cancer

**DOI:** 10.3389/fphar.2023.1159712

**Published:** 2023-05-22

**Authors:** Jiawei Li, Fangfang Li, Yuqing Zhao, Dan Jin

**Affiliations:** ^1^ Immunology Biology Key Laboratory, Yanbian University, Yanji, China; ^2^ Key Laboratory of Natural Medicines of the Changbai Mountain, Ministry of Education, Yanbian University, Yanji, China

**Keywords:** AD-1, colorectal cancer, network pharmacology, cell apoptosis, PI3K-Akt signaling pathway

## Abstract

20 (R)-25-methoxyl-dammarane-3β, 12β, 20-triol (AD-1), a novel ginsenoside isolated from stem and leaf of Panax Notoginseng, has anticancer activity against a variety of malignant tumors. However, the pharmacological mechanism of AD-1 on colorectal cancer (CRC) remains unclear. The purpose of this study was to verify the potential mechanism of action of AD-1 against CRC through network pharmacology and experiments. A total of 39 potential targets were obtained based on the intersection of AD-1 and CRC targets, and key genes were analyzed and identified from the PPI network using Cytoscape software. 39 targets were significantly enriched in 156 GO terms and 138 KEGG pathways, among which PI3K-Akt signaling pathway was identified as one of the most enriched pathways. Based on experimental results, AD-1 can inhibit the proliferation and migration of SW620 and HT-29 cells, and induce their apoptosis. Subsequently, the HPA and UALCAN databases showed that PI3K and Akt were highly expressed in CRC. AD-1 also decreased the expressions of PI3K and Akt. In summary, these results suggest that AD-1 can play an anti-tumor role by inducing cell apoptosis and regulating PI3K-Akt signaling pathway.

## 1 Introduction

Colorectal cancer (CRC) is the third most common cancer in the world, ranking the third in incidence and the second in mortality (Sung et al., 2021). Despite great progress in the treatment of CRC, the survival rate of patients with advanced CRC is still low due to poor prognosis (Gbolahan et al., 2019; Miller et al., 2019). In addition, chemotherapy has certain side effects, which can lead to drug resistance, while targeted therapy also has a certain burden of symptoms (Veenstra et al., 2018). Therefore, the development of effective anti-CRC drugs is particularly important.

Ginsenosides are the main active components of ginseng, with anti-tumor, anti-inflammatory and other pharmacological effects (Wei et al., 2020; Ren et al., 2021). 20 (R)-25-methoxyl-dammarane-3β, 12β, 20-triol (AD-1) is a novel saponin derivative isolated from the hydrolyzed saponins from the stem and leaf of panax notoginseng. It has been reported that AD-1 not only plays an anti-hepatic fibrosis role (Han et al., 2018; Li et al., 2022), but also can inhibit the activity of gastric cancer and lung cancer (Zhang et al., 2013; Zhao et al., 2016). Zhang et al. (2013) pointed out that AD-1 induced lung cancer cell arrest and apoptosis by activating p38 MAPK signaling pathway. However, the anti-CRC effect of AD-1 has not been studied. Therefore, it would be interesting to explore the roles and potential mechanisms of AD-1 in CRC.

**FIGURE 1:**
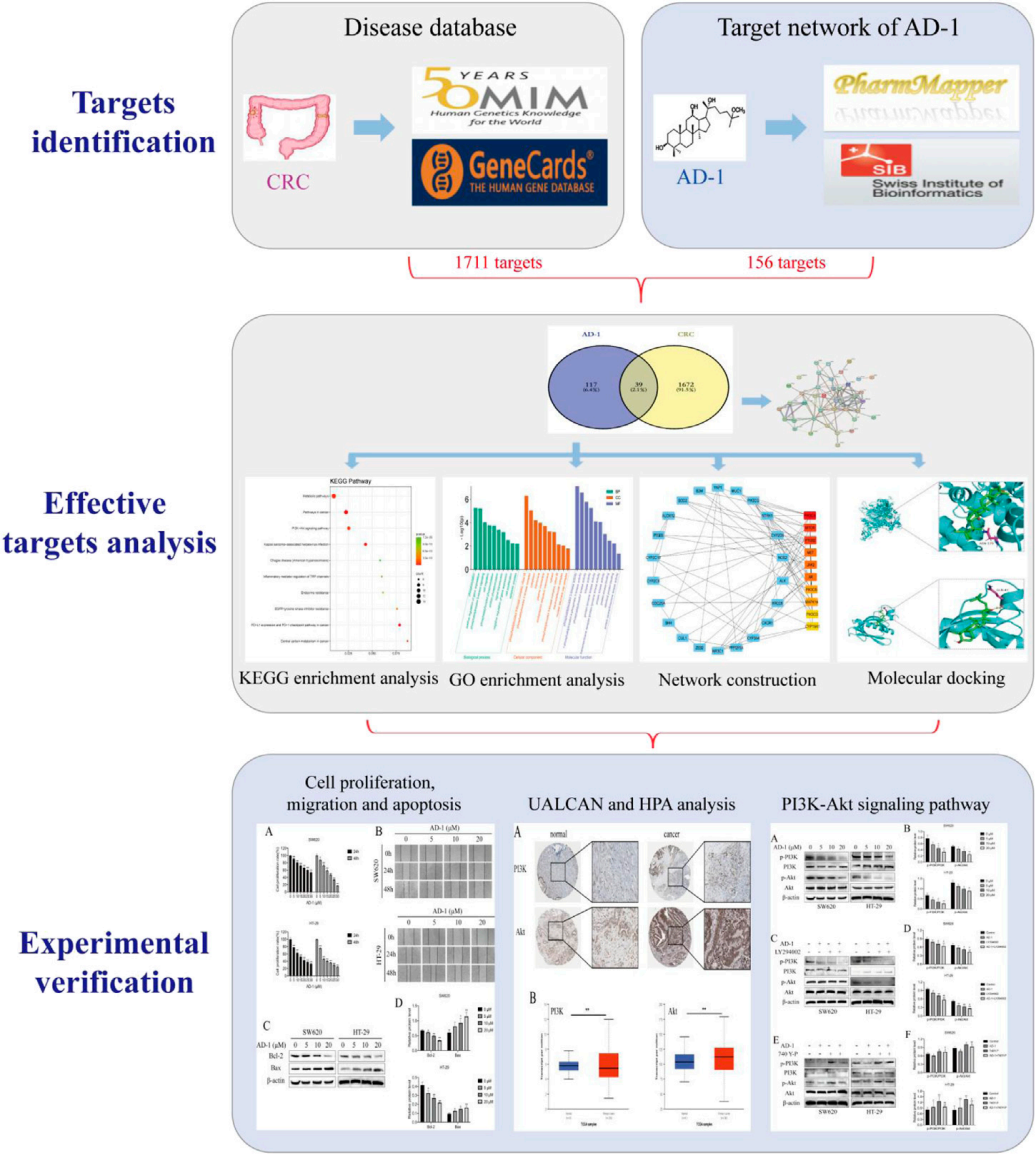
Schematic flowchart of the study design.

Network pharmacology is a systematic approach that combines systems biology and bioinformatics to elucidate the potential mechanisms of action of drugs (Nogales et al., 2022). In this study, we used network pharmacology to predict potential targets of AD-1 and CRC, and screened central molecules by PPI and enrichment analysis. Finally, the molecular mechanism of the above phenomenon was verified by *in vitro* experiments. Figure 1 depicts this workflow study.

## 2 Materials and methods

### 2.1 Potential targets of AD-1

Through PharmMapper (http://lilab.ecust.edu.cn/pharmmapper/) (Wang et al., 2017), the Swiss Target Prediction Screening (http://www.swisstargetprediction.ch/) (Daina et al., 2019) of AD-1 database of potential targets. Use UniProt (https://www.uniprot.org/) to improve the UniProt ID and gene name of the target.

### 2.2 Identification of CRC-related gene targets

Use “colorectal cancer” as the keyword in the online Mendelian inheritance in man (OMIM, https://omim.org/) (Amberger et al., 2015) database, GeneCards (https://www.genecards.org/) (Rebhan et al., 1997) database (correlation score of 20 or higher) to retrieve the CRC related targets. The results of different databases were integrated to delete duplicate target genes and finally obtain potential CRC-related targets.

### 2.3 Target prediction of AD-1 anti-CRC

Potential drug targets and CRC related genes were uploaded to VENNY2.1 website (https://bioinfogp.cnb.csic.es/tools/venny/index.html), to determine the cross genes, both the common targets for AD-1 candidate targets for the treatment of CRC.

### 2.4 Analysis of protein-protein interaction (PPI) network

The cross genes obtained in 2.3 were imported into the STRING database (https://string-db.org/) to construct the PPI network. Cytoscape (version 3.8.2) software and CytoHubba plug-ins are then used for visual analysis to sort the nodes in the network according to the algorithm of degree and select the top 10 core genes.

### 2.5 Gene ontology (GO) and kyoto encyclopedia of genes and genomes (KEGG) enrichment analysis

We will cross gene into the database for annotation, visualization, and integrated discovery (DAVID) (https://david.ncifcrf.gov/) (Sherman et al., 2022) to GO enrichment analysis, the database from a biological process (BP), cell composition (CC) and molecular function (MF) evaluated the goal of participation. In addition, we used KOBAS 3.0 database (http://kobas.cbi.pku.edu.cn/) (Bu et al., 2021) KEGG pathway enrichment. Then will GO and KEGG results import bioinformatics website (http://www.bioinformatics.com.cn/) for annotation and visualization.

### 2.6 Molecular docking verification

The AD-1 structure was imported into ChemBio3D Ultra 14.0 software to minimize energy and obtain the 3D structure, which were further inputted into AutodockTools-1.5.6 and saved as “pdbqt” format. The protein 3D structure was obtained from the Protein Data Bank (PDB, http://www.rcsb.org/) and then imported into Pymol 2.3.0 to remove the protein crystallinity water, the original ligand, etc. After that, the protein structure was imported into AutoDocktools (v1.5.6) and saved as “pdbqt” format. Finally, Autodock 4.26 was used for molecular docking, and the results were visualized with PyMOL2.3.0.

### 2.7 Chemicals

AD-1 (isolated from Panax notoginseng), colorless crystals, the purity of 99.9%. Its molecular formula was ensured to be C31H56O4 by 13C NMR and ESI MS [m/z 493 (M + H)^+^ and 515 (M + Na)^+^], which was ascertained through HRE-IMS (m/z 515.4064 calcd for C31H56O4Na, 515.4076). Finally, HMBC spectrum analysis, the construction of the AD-1 was confirmed as 20 (R)-25-methoxyl-dammarane-3β, 12β, 20-triol. And then, AD-1 was dissolved in dimethyl subunit DMSO (Sigma-Aldrich, MO, United States), then divided into several centrifuge tubes and stored at −20°C for subsequent use.

### 2.8 Cell culture

The SW620 and HT-29, human CRC cell lines, were purchased from the American Type Culture Collection. These cells were cultured in DMEM medium (Biological Industries, BeitHaEmek, Israel) including 10% FBS (Biological Industries, BeitHaEmek, Israel) at 37°C in a 5% CO_2_.

### 2.9 Cell viability assay

The cells were inoculated into 96-well plates at 1 × 10^4^ cells/well and cultured for 24 h. Subsequent incubations were performed with different concentrations of AD-1. After 24 h or 48 h, 10 μL CCK-8 solution (Beyotime Biotechnology, Jiangsu, China) was added to each well. The corresponding absorbance was measured after 2 h.

### 2.10 Wound healing assay

The cells were implanted into a 6-well plate of 2 × 10^6^ cells/well. Monolayer cells were scraped with a sterile pipette to form the wound, washed with PBS for 3 times, and cultured with AD-1 solution for 0 h, 24 h, and 48 h, then imaging examination of the wound was performed.

### 2.11 UALCAN and human protein atlas (HPA) database analysis

TCGA dataset was used to compare the expression of PI3K-Akt signaling pathway in normal and CRC tissues. Examples of proteins found in the HPA database that showed a trend of differential expression in normal and CRC tissues. The above data analysis from the UALCAN (http://ualcan.path.uab.edu/index.html) and HPA (http://www.proteinatlas.org) database.

### 2.12 Western blot

CRC cells (2 × 10^6^ cells/well) were inoculated in 6-well plates for 24 h and treated with different concentrations of AD-1 for 48 h. Subsequently, protein were isolated and quantified from SW620 and HT-29 cells. The required proteins were isolated on SDS-PAGE gels and shifted to PVDF membrane, closed for 1 h. Subsequent, cultivated with antibodies including Bax, Bcl-2 (Abcam, Cambridge, UK), p-PI3K, PI3K, p-Akt, Akt, and β-actin (Cell Signaling Techno-logy, Boston, MA, United States) for 24 h. The corresponding secondary antibodies were used to incubate the membrane for 1 h (Aleuronic, Beijing, China). The strip films were incubated by ECL chemiluminescence (Everbright, San Ramon, CA, United States). Finally, the membranes were developed and imaged.

### 2.13 Statistical analysis

All experiments were repeated three times, and the data are presented as the mean ± SD. Group means were compared using a *t*-test. *p* < 0.05 was considered statistically significant.

## 3 Results

### 3.1 Screening of potential targets of AD-1 against CRC

**FIGURE 2:**
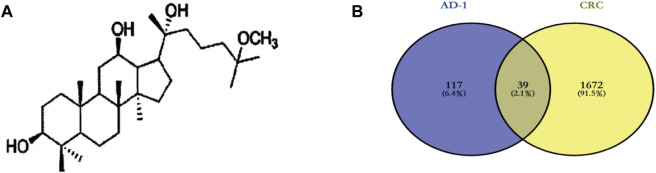
Targets screening involved in AD-1 for treating CRC. **(A)** The chemical structure of AD-1. **(B)** Thirty-nine overlapping target proteins between CRC-related genes and targets of AD-1.

The chemical structure of AD-1 is shown in Figure 2A. The molecular structure of AD-1 was uploaded to PharmMapper database and Swiss Target Prediction database and 156 potential targets of AD-1 were identified. In addition, a total of 1711 CRC-related targets were obtained from the OMIM database and GeneCards database. AD-1 targets and CRC-related genes were then uploaded to VENNY2.1 to generate a Venn diagram (Figure 2B). There were 39 intersections have been identified as potential candidate targets for AD-1 against CRC (Table 1).

**TABLE 1:** Potential target genes of AD-1 for CRC.

No.	Sybol	Uniprot ID	No.	Sybol	Uniprot ID
1	SHH	Q15465	21	NR3C1	P04150
2	PIK3CB	P42338	22	MTOR	P42345
3	PIK3CD	O00329	23	PTK6	Q13882
4	PIK3CG	P48736	24	CDC25A	P30304
5	PIK3CA	P42336	25	ALK	Q9UM73
6	CYP19A1	P11511	26	YAP1	P19880
7	MAPK14	Q16539	27	SDHA	Q9YHT1
8	PTGES	P35354	28	S100A11	P24480
9	MET	P08581	29	POT1	Q9NUX5
10	CASR	P41180	30	SOD2	P04179
11	PTGS2	P35354	31	PPP2R1A	P30153
12	NTRK1	P04629	32	B2M	P61769
13	JAK2	O60674	33	ALOX12	P18054
14	CXCR1	P25024	34	PEBP1	P13696
15	CYP2D6	P10635	35	XRCC6	P12956
16	CYP2C9	P11712	36	RHO	P02699
17	CYP3A4	P08684	37	MUC1	P15941
18	CYP2C19	P33261	38	ZEB2	O60315
19	NOS2	P35228	39	CUL1	Q13616
20	AR	P10275			

### 3.2 PPI network analysis and screening of key targets

**FIGURE 3:**
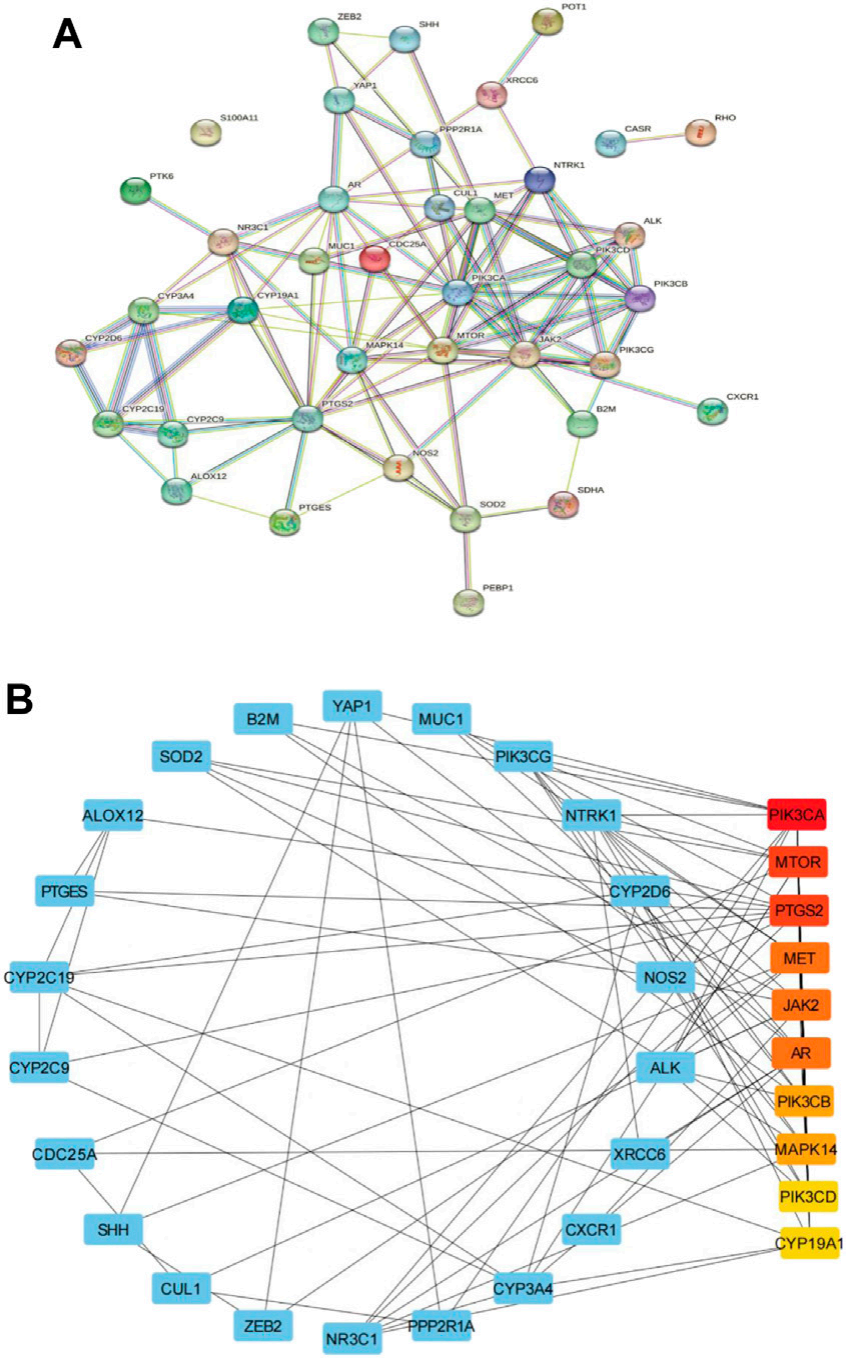
Construction of PPI network. **(A)** PPI network of 39 cross-target. **(B)** The first 10 key genes generated by **(A)**. The darker the node color (red), the stronger the connection.

In order to further explore the relationship between cross genes, they were uploaded to STRING database to construct PPI network, which contained 39 nodes and 109 edges (Figure 3A). Then, we upload the PPI network to Cytoscape software for visualization, and utilize CytoHubba plug-ins, the top 10 key genes (ranked from high to low) were screened by degree method: PIK3CA, MTOR, PTGS2, MET, JAK2, AR, PIK3CB, MAPK14, PIK3CD, and CYP19A1 (Figure 3B; Table 2). PIK3CA is the topmost node with a connection of 32°, which may play an important role in the anti-CRC mechanism of AD-1.

**TABLE 2:** Top 10 core proteins information.

Uniprot ID	Sybol	Protein names	Degree
P42336	PIK3CA	PI3-kinase p110-alpha subunit	32
P42345	MTOR	Serine/threonine-protein kinase mTOR	30
P35354	PTGS2	Cyclooxygenase-2	30
P08581	MET	Hepatocyte growth factor receptor	24
O60674	JAK2	Tyrosine-protein kinase JAK2	24
P10275	AR	Androgen Receptor	24
P42338	PIK3CB	PI3-kinase p110-beta subunit	18
Q16539	MAPK14	MAP kinase p38 alpha	18
O00329	PIK3CD	PI3-kinase p110-delta subunit	16
P11511	CYP19A1	Cytochrome P450 19A1	16

### 3.3 GO and KEGG pathway enrichment analysis

**FIGURE 4:**
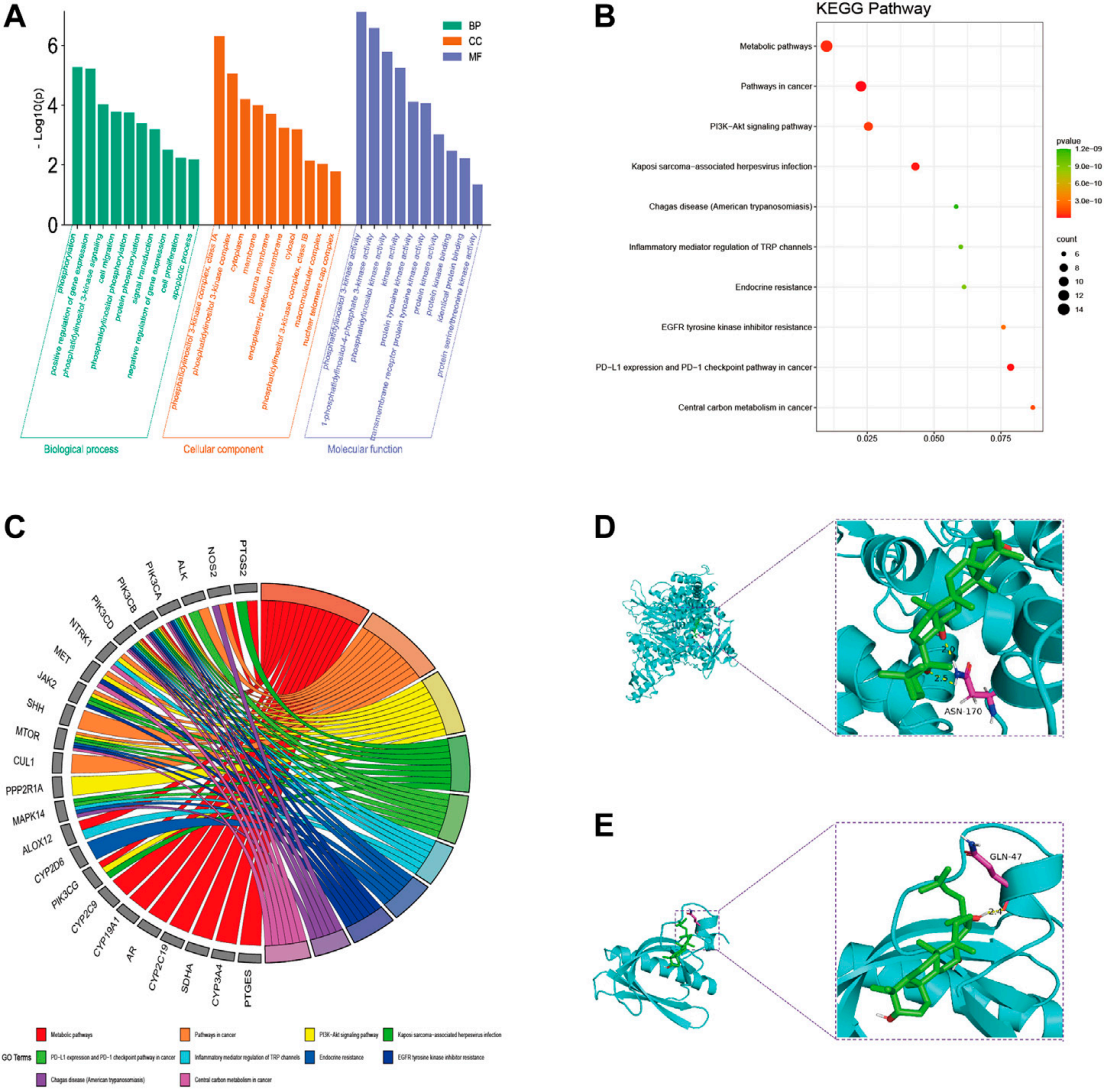
GO and KEGG pathway analysis of intersecting genes. **(A)** GO enrichment analysis, including BP, CC, and MF. **(B)** Bubble diagram of KEGG pathway enrichment. **(C)** GO chord diagram. **(D, E)** The docking mode of AD-1 binding with target proteins; **(D)** PI3K, **(E)** Akt.

DAVID database was used to analyze the GO analysis of 39 cross genes. The 39 cross genes of AD-1 against CRC were significantly enriched in 106 BPs, 16 CCs, and 34 MFs (*p* < 0.05). As shown in Figure 4A, BP enrichment analysis showed that AD-1 may be involved in the regulation of signal transduction, protein phosphorylation, cell proliferation, cell migration and apoptotic process. CC enrichment analysis showed that AD-1 mainly plays a role in cytoplasm, membrane and cytosol. MF enrichment analysis suggested that the antitumor effect of AD-1 might be related to its effects on kinase activity, protein kinase activity, protein kinase binding and identical protein binding. In addition, KEGG pathway analysis was performed on KOBAS database and 138 pathways were screened (*p* < 0.05). It was found that metabolic pathways, pathways in cancer and PI3K-Akt signaling pathway were the top three significant pathways, with 14, 12 and 9 cross-genes, respectively (Figures 4B, C). PI3K-Akt signaling pathway plays a crucial role in regulating the development of tumor cells. Furthermore, PI3K-Akt signaling pathway is activated in malignant tumors and can directly regulate the proliferation, migration and apoptosis of tumor cells, which is a potential therapeutic target. Tan et al. (2021) pointed out that the PI3K-Akt signaling pathway is important in CRC, and found that promethazine promoted apoptosis by inhibiting the activation of PI3K-Akt signaling pathway in CRC cells. Next, we used molecular docking simulations to predict the binding capacity of AD-1 to PI3K and Akt. A lower binding energy indicates a more efficient and stable interaction between the compound and the receptor. As shown in Figures 4D, E, the binding energies of AD-1 to PI3K and Akt were −8.4 and −6.2 kcal/mol, respectively, indicating their good binding activities (Table 3).

**TABLE 3:** Result of molecular docking.

Targets	PDB	Binding energy (kcal/mol)
Akt	1UNQ	−6.2
PI3K	4JPS	−8.4

### 3.4 AD-1 inhibited cell proliferation and migration of CRC cells

**FIGURE 5:**
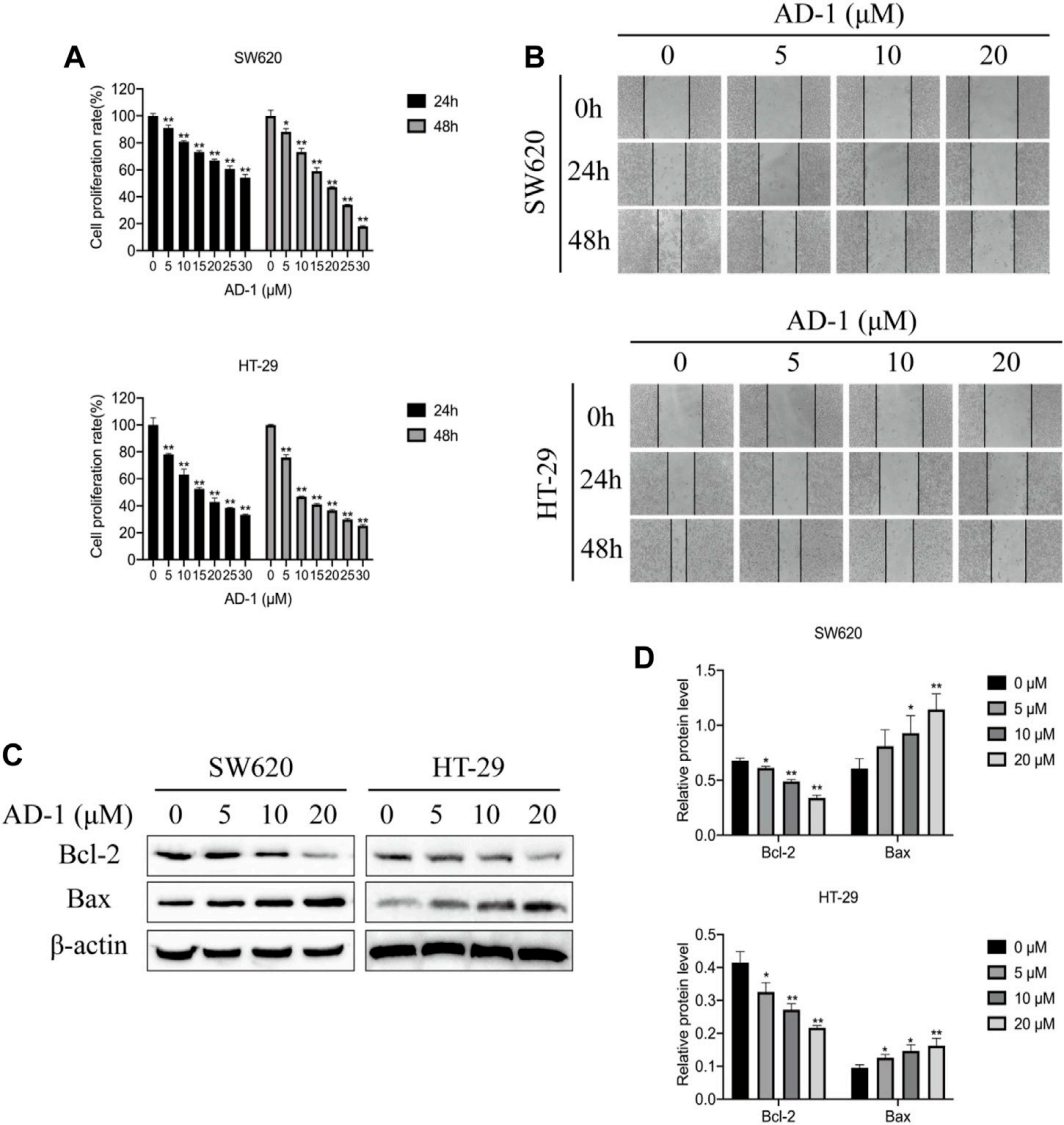
AD-1 inhibited CRC cell proliferation, migration and induced apoptosis. **(A)** SW620 and HT-29 cell proliferation was measured after treatment with AD-1 at different concentrations for 24 and 48 h. **(B)** The ability of SW620 and HT-29 cells to migrate was examined by wound healing assay. **(C,D)** The protein expressions of Bcl-2, Bax and β-actin were evaluated by western blot. ^*^: vs. the control group, ^*^ *p* < 0.05, ^**^ *p* < 0.01.

To verify the anti-proliferation effect of AD-1 on CRC cells, CCK8 was used to detect the proliferation ability of SW620 and HT-29 cells treated with AD-1 for 24 and 48 h. The results showed that AD-1 significantly inhibited the proliferation of SW620 and HT-29 cells. The IC50 values of SW620 and HT-29 cells treated with AD-1 for 24 h were 36.22 µM, 15.52 µM, respectively. The IC50 values of SW620 and HT-29 cells treated with AD-1 for 48 h were 18.65 µM, 11.07 µM, respectively (Figure 5A). Therefore, 0, 5, 10, 20 µM concentration was selected for 48 h for subsequent study. In addition, wound healing assay was performed to evaluate the effect of AD-1 on the migration ability of SW620 and HT-29 cells. The results showed that with the increase of AD-1 concentration, the migration ability of SW620 and HT-29 cells was significantly inhibited (Figure 5B).

### 3.5 AD-1 induced apoptosis in CRC cells

Bcl-2 family proteins, including Bcl-2 and Bax, are key regulatory genes that initiate apoptosis. To detect the effect of AD-1 on apoptosis of SW620 and HT-29 cells, the levels of apoptosis-related proteins were detected by western blot. The results showed that AD-1 significantly decreased the expression of Bcl-2 protein, but increased the expression of Bax protein in a dose-dependent manner (Figures 5C, D). Besides, AD-1 dramatically increased the percentage of apoptosis cells (Supplementary Figure S1). These results suggested that AD-1 could efficiently induce apoptosis of CRC cells.

### 3.6 PI3K-Akt signaling pathway was highly expressed in colon tissues

**FIGURE 6:**
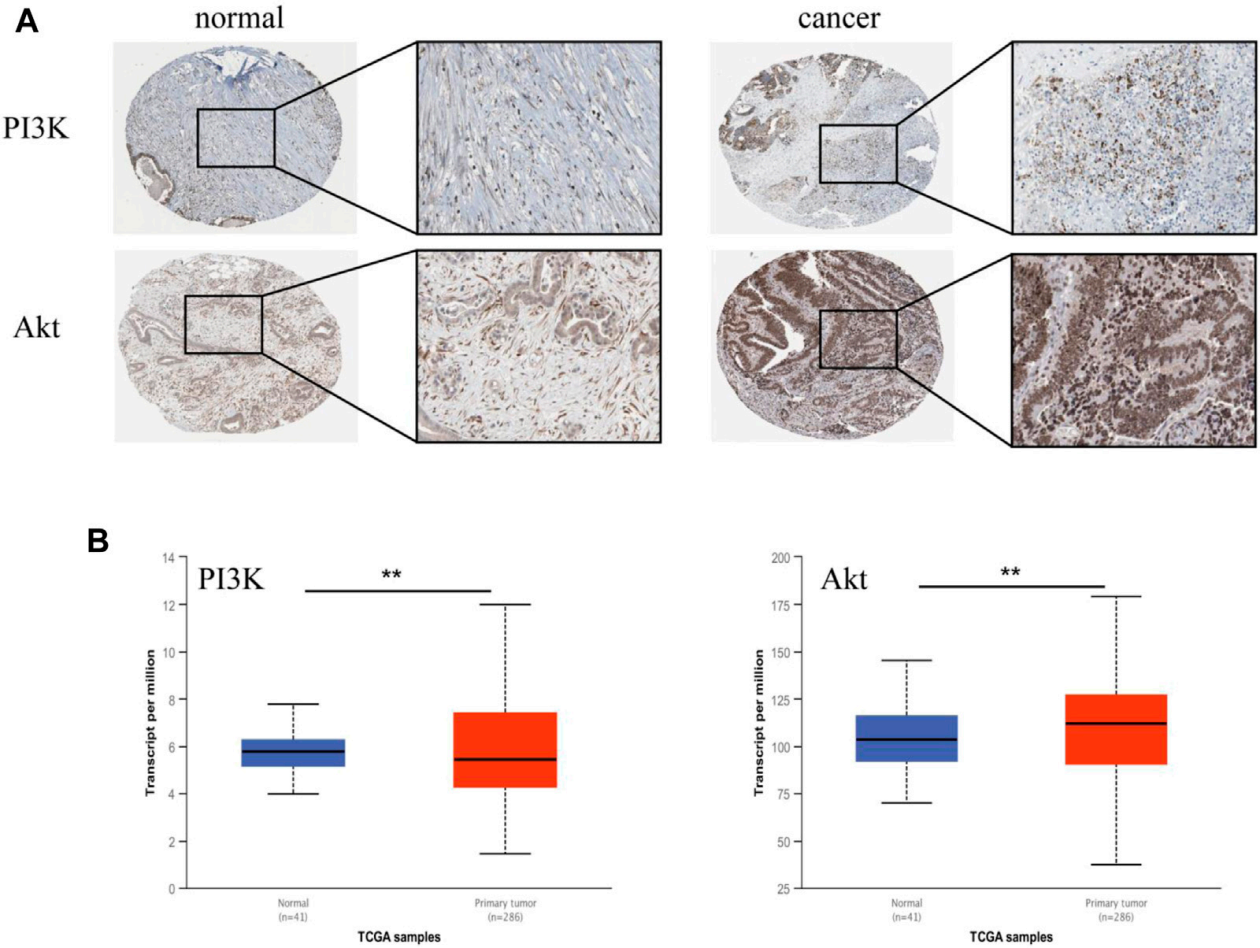
Validation of PI3K and Akt genes in HPA and UALCAN databases. **(A)** The representative immunohistochemical images of PI3K and Akt in normal tissue and CRC tissue. The right picture is a magnified image corresponding to (×400). **(B)** The expression of PI3K and Akt in UALCAN database. Tumors are shown in red and normal tissues in blue. ^*^: vs. the control group, ^*^ *p* < 0.05, ^**^ *p* < 0.01.

These results indicated that AD-1 could significantly inhibit cell growth and migration, and induce cell apoptosis. According to the results of network pharmacological analysis, the PI3K-Akt signaling pathway is involved in the anti-CRC mechanism of AD-1. To test this hypothesis, we analyzed the correlation between the PI3K-Akt signaling pathway and CRC using the HPA and UALCAN databases. We analyzed PI3K and Akt expression at the protein level in normal and CRC tissues using data from the HPA. Notably, CRC tissue was highly positive in multiple patients, primarily in the cytoplasm/membrane (Figure 6A). In addition, mRNA expressions of PI3K and Akt in CRC tissues were significantly higher than those in normal tissues (Figure 6B). It is suggested that PI3K and Akt are highly expressed in CRC.

### 3.7 AD-1 inhibited the activation of the PI3K-Akt signaling pathway in CRC cells

**FIGURE 7:**
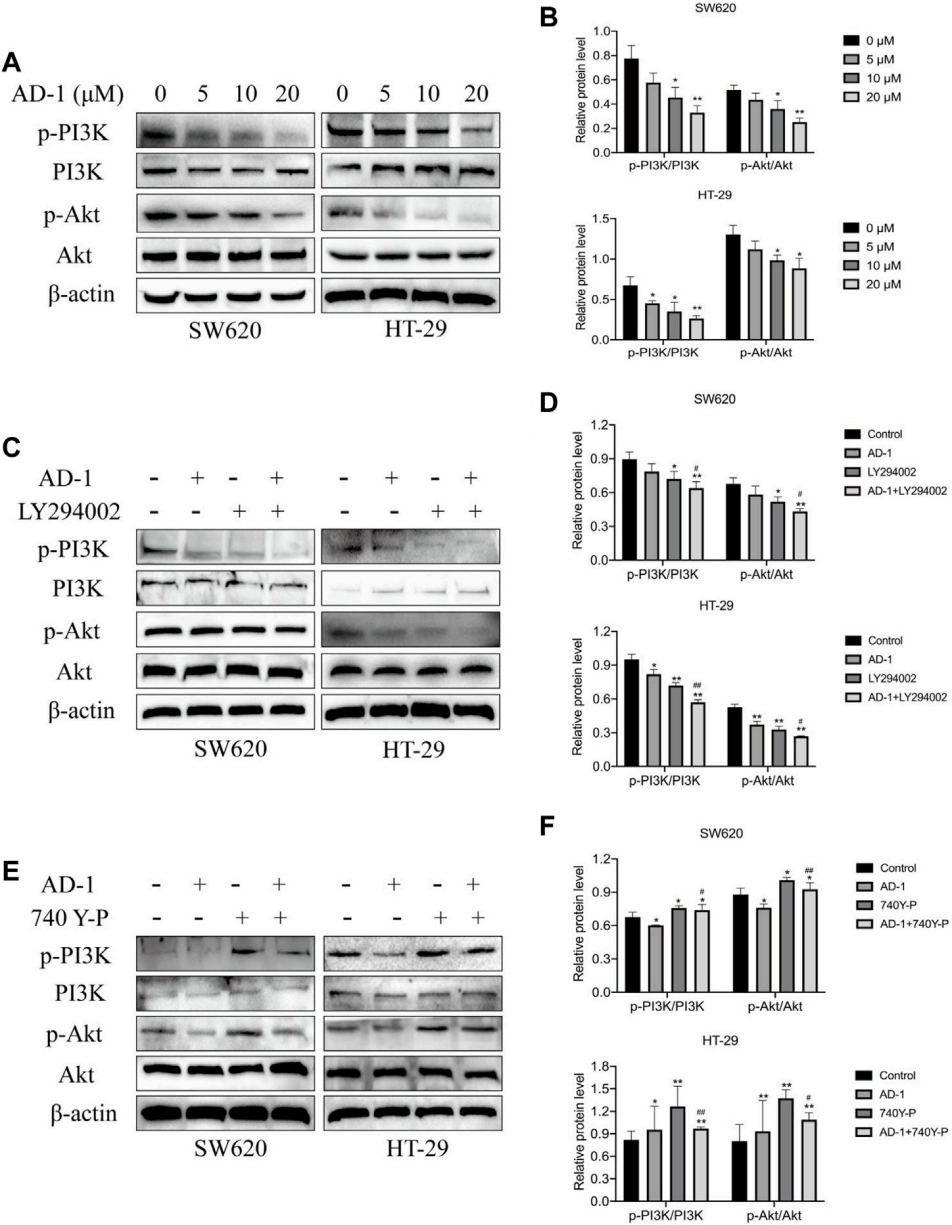
AD-1 inhibits the activation of PI3K-Akt signaling pathway. **(A, B)** The protein expression of p-PI3K, PI3K, p-Akt, and Akt was detected by western blot. **(C, D)** CRC cells were pretreated with PI3K inhibitor LY294002, the effects on PI3K-Akt signaling pathway were measured. **(E, F)** CRC cells were pretreated with PI3K activator 740Y-P, the effects on PI3K-Akt signaling pathway were measured. ^*^: vs. the control group, ^*^ *p* < 0.05, ^**^ *p* < 0.01. ^#^:vs. the LY294002 group or 740Y-P group, ^#^ *p* < 0.05, ^##^ *p* < 0.01.

To further test this hypothesis, we evaluated the expression levels of PI3K, p-PI3K, Akt, and p-Akt in SW620 and HT-29 cells after AD-1 intervention. The results showed that AD-1 significantly inhibited the phosphorylation levels of PI3K and Akt (Figures 7A, B), indicating that the PI3K-Akt pathway was inhibited by AD-1 in CRC cells. Next, we further explored the role of the PI3K-Akt signaling pathway in AD-1 mediated regulation of CRC. We used PI3K inhibitor LY294002 (20 μM) to block the activity of PI3K-Akt, and PI3K activator 740Y-P (5 μM) to activate the activity of PI3K-Akt. We found that the inhibitor not only inhibited the activation of PI3K-Akt pathway, but also promoted the inhibition of this pathway by AD-1 (Figures 7C, D). The activation of PI3K-Akt pathway induced by PI3K activator 740Y-P can be reversed by the addition of AD-1 (Figures 7E, F). These results indicate that AD-1 is involved in the proliferation of CRC cells by regulating the PI3K-Akt signaling pathway.

## 4 Discussion

In recent years, more and more attention has been paid to the application of Chinese herbal medicine, which has become a new trend in tumor treatment. Many studies have shown that AD-1 can inhibit cell growth and promote apoptosis in tumor cells such as gastric cancer, lung cancer, breast cancer (Wang et al., 2012; Zhang et al., 2013; Zhao et al., 2016). AD-1 has the characteristics of low toxicity and multi-target prevention and treatment of CRC. The theoretical concept based on network pharmacology is similar to the multi-component and multi-target of traditional Chinese medicine, which is an effective and clear method to reveal the targets of drugs and diseases. Therefore, this study aimed to elucidate the effects of AD-1 on CRC and further verify the mechanism predicted by network pharmacology using *in vitro* experiments.

First, we identified 39 crossover targets from 156 potential AD-1 targets and 1711 CRC-related targets. Then, PPI network was constructed and 10 hub genes were found. These genes are involved in the regulation of signal transduction, protein phosphorylation, cell proliferation, cell migration and apoptosis process. Based on KEGG pathway analysis, PI3K-Akt pathway was one of the most important pathways with 9 enriched cross genes. Molecular docking analysis confirmed that AD-1 had a high binding potential with PI3K and Akt. Therefore, we speculate that AD-1 may affect CRC cell proliferation, migration, and apoptosis by binding to some core proteins in the PI3K-Akt signaling pathway.

Based on the above network pharmacological research, we conducted the verification of relevant experiments. In our study, AD-1 showed some anti-proliferative effects on CRC cells by cell viability measurements. In addition, AD-1 inhibits CRC cell migration. The Bcl-2 protein family is closely related to the mitochondrial apoptosis pathway, and Bax is a regulatory factor of Bcl-2, which can change the changes of mitochondrial permeability and activate apoptosis-related proteins (Hird et al., 2019). AD-1 can induce apoptosis of a variety of cancer cells, such as BGC-823, SGC-7901, MSN-28 cells (Zhao et al., 2016), lung cell carcinoma A549 and H292 cells (Zhang et al., 2013), and breast cancer MCF7 and MDA-MB-231 cells (Wang et al., 2012). Here, we assessed whether AD-1 induced apoptosis in CRC cells. The results showed that AD-1 significantly reduced the protein expression of Bcl-2 and increased the protein level of Bax, suggesting that AD-1 could induce apoptosis of SW620 and HT-29 cells and regulate the death of CRC cells. In conclusion, AD-1 has anti-proliferation and pro-apoptosis effects on CRC cells, and its specific mechanism is worthy of further study.

In recent years, the PI3K-Akt signaling pathway is widely present in a variety of cell signal transduction pathways, and is one of the hot spots in the research of malignant tumors (Dong et al., 2018). PI3K is an intracellular phosphatidyl inositol kinase composed of a catalytic subunit p110 and a regulatory subunit p85. PI3K can be activated by receptors on the cell surface to produce the second messenger phosphatidylinosite-3, 4, 5-triphosphate (PIP3), which can further activate downstream effector factors such as Akt and participate in the regulation of cell proliferation, migration, apoptosis and other signal transduction (Vanhaesebroeck et al., 2010; Ruicci et al., 2019). A large number of studies have shown that the occurrence and development of various diseases, including cancer, may be related to the abnormal activation of this pathway, such as colon cancer, non-small cell lung cancer and prostate cancer (Jiang et al., 2021; Zhu et al., 2021; Alharbi et al., 2022). In this study, we used network pharmacology to predict the potential anti-CRC mechanism of AD-1. The results showed that PI3K-Akt pathway has 9 enriched cross genes and central gene PIK3CA, suggesting that this pathway may be a key pathway involved in the anti-CRC mechanism of AD-1. To test this hypothesis, we searched the UALCAN and HPA databases and found that PI3K and Akt were highly expressed in CRC. In addition, the protein expressions of PI3K, p-PI3K, Akt, and p-Akt were detected by western blot. These results are consistent with our findings above that AD-1 significantly reduces the phosphorylation of PI3K and Akt, suggesting that PI3K-Akt may be an effective phosphorylation pathway.

In conclusion, our results suggest that AD-1 inhibits SW620 and HT-29 cell proliferation and promotes apoptosis by regulating the PI3K-Akt signaling pathway. We believe that AD-1 may be a promising therapeutic agent for CRC.

## Data Availability

The original contributions presented in the study are included in the article/Supplementary Material, further inquiries can be directed to the corresponding authors.

## References

[B1] Alharbi K. S. Shaikh M. A. J. Almalki W. H. Kazmi I. Al-Abbasi F. A. Alzarea S. l. (2022). PI3K/Akt/mTOR pathways inhibitors with potential prospects in non-small-cell lung cancer. J. Environ. Pathol. Toxicol. Oncol. 41, 85–102. 10.1615/JEnvironPatholToxicolOncol.2022042281 36374963

[B2] Amberger J. S. Bocchini C. A. Schiettecatte F. Scott A. F. Hamosh A. (2015). OMIM.org: Online Mendelian Inheritance in Man (OMIM®), an online catalog of human genes and genetic disorders. Nucleic. acids. Res. 43, D789–D798. 10.1093/nar/gku1205 25428349 PMC4383985

[B3] Bu D. C. Luo H. T. Huo P. P. Wang Z. H. Zhang S. He Z. H. (2021). KOBAS-I: Intelligent prioritization and exploratory visualization of biological functions for gene enrichment analysis. Nucleic. acids. Res. 49, W317–W325. 10.1093/nar/gkab447 34086934 PMC8265193

[B4] Daina A. Michielin O. Zoete V. (2019). SwissTargetPrediction: Updated data and new features for efficient prediction of protein targets of small molecules. Nucleic. acids. Res. 47, W357–W364. 10.1093/nar/gkz382 31106366 PMC6602486

[B5] Dong P. P. Hao F. M. Dai S. F. Tian L. (2018). Combination therapy Eve and Pac to induce apoptosis in cervical cancer cells by targeting PI3K/AKT/mTOR pathways. J. Recept. Signal. Transduct. Res. 38, 83–88. 10.1080/10799893.2018.1426610 29369007

[B6] Gbolahan O. O'Neil B. (2019). Update on systemic therapy for colorectal cancer: Biologics take sides. Transl. Gastroenterol. Hepatol. 4, 9. 10.21037/tgh.2019.01.12 30976712 PMC6414333

[B7] Han X. Song J. Lian L. H. Yao Y. L. Shao D. Y. Fan Y. (2018). Ginsenoside 25-OCH3-PPD promotes activity of LXRs to ameliorate P2X7R-mediated NLRP3 inflammasome in the development of hepatic fibrosis. J. Agric. Food. Chem. 66, 7023–7035. 10.1021/acs.jafc.8b01982 29929367

[B8] Hird A. W. Tron A. E. (2019). Recent advances in the development of Mcl-1 inhibitors for cancer therapy. Pharmacol. Ther. 198, 59–67. 10.1016/j.pharmthera.2019.02.007 30790641

[B9] Jiang T. Wang H. Y. Liu L. Y. Song H. Zhang Y. Wang J. Q. (2021). CircIL4R activates the PI3K/AKT signaling pathway via the miR-761/TRIM29/PHLPP1 axis and promotes proliferation and metastasis in colorectal cancer. Mol. Cancer. 20, 167. 10.1186/s12943-021-01474-9 34922544 PMC8684286

[B10] Li T. Chen Y. Li Y. Chen G. Zhao Y. Q. Su G. Y. (2022). Antifibrotic effect of AD-1 on lipopolysaccharide-mediatedfibroblast injury in L929 cells andbleomycin-induced pulmonaryfibrosis in mice. Food. Funct. 13, 7650–7665. 10.1039/d1fo04212b 35735105

[B11] Miller K. D. Nogueira L. Mariotto A. B. Rowland J. H. Yabroff K. R. Alfano C. M. (2019). Cancer treatment and survivorship statistics, 2019. Ca. Cancer. J. . Clin. 69, 363–385. 10.3322/caac.21565 31184787

[B12] Nogales C. Mamdouh Z. M. List M. Kiel C. Casas A. I. Schmidt H. H. H. W. (2022). Network pharmacology: Curing causal mechanisms instead of treating symptoms. Trends. Pharmacol. Sci. 43, 136–150. 10.1016/j.tips.2021.11.004 34895945

[B13] Rebhan M. Chalifa-Caspi V. Prilusky J. Lancet D. (1997). GeneCards: Integrating information about genes, proteins and diseases. Trends. Genet. 13, 163. 10.1016/s0168-9525(97)01103-7 9097728

[B14] Ren B. Feng J. P. Yang N. Guo Y. J. Chen C. Qin Q. (2021). Ginsenoside Rg3 attenuates angiotensin II-induced myocardial hypertrophy through repressing NLRP3 inflammasome and oxidative stress via modulating SIRT1/NF-κB pathway. Int. Immunopharmacol. 98, 107841. 10.1016/j.intimp.2021.107841 34153662

[B15] Ruicci K. M. Plantinga P. Pinto N. Khan M. L. Stecho W. Dhaliwal S. S. (2019). Disruption of the RICTOR/mTORC2 complex enhances the response of head and neck squamous cell carcinoma cells to PI3K inhibition. Mol. Oncol. 13, 2160–2177. 10.1002/1878-0261.12558 31393061 PMC6763779

[B16] Sherman B. T. Hao M. Qiu J. Jiao X. Baseler M. W. Lane H. C. (2022). David: A web server for functional enrichment analysis and functional annotation of gene lists (2021 update). Nucleic. acids. Res. 50, W216–W221. 10.1093/nar/gkac194 35325185 PMC9252805

[B17] Sung H. Ferlay J. Siegel R. L. Laversanne M. Soerjomataram I. Jemal A. (2021). Global cancer statistics 2020: Globocan estimates of incidence and mortality worldwide for 36 cancers in 185 countries. Ca. Cancer. J. Clin. 71, 209–249. 10.3322/caac.21660 33538338

[B18] Tan X. Y. Gong L. Y. Li X. Y. Zhang X. Y. Sun J. H. Luo X. H. (2021). Promethazine inhibits proliferation and promotes apoptosis in colorectal cancer cells by suppressing the PI3K/AKT pathway. Biomed. Pharmacother. 143, 112174. 10.1016/j.biopha.2021.112174 34560542

[B19] Vanhaesebroeck B. Guillermet-Guibert J. Graupera M. Bilanges B. (2010). The emerging mechanisms of isoform-specific PI3K signalling. Nat. Rev. Mol. Cell. Biol. 11, 329–341. 10.1038/nrm2882 20379207

[B20] Veenstra C. M. Krauss J. C. (2018). Emerging systemic therapies for colorectal cancer. Clin. Colon. Rectal. Surg. 31, 179–191. 10.1055/s-0037-1602238 29720904 PMC5929883

[B21] Wang W. Zhang X. Qin J. J. Voruganti S. Ashok Nag S. Wang M. H. (2012). Natural product ginsenoside 25-OCH3-PPD inhibits breast cancer growth and metastasis through down-regulating MDM2. Plos. One. 7, e41586. 10.1371/journal.pone.0041586 22911819 PMC3402429

[B22] Wang X. Shen Y. H. Wang S. W. Li S. L. Zhang W. L. Liu X. F. (2017). PharmMapper 2017 update: A web server for potential drug target identification with a comprehensive target pharmacophore database. Nucleic. acids. Res. 45, W356–W360. 10.1093/nar/gkx374 28472422 PMC5793840

[B23] Wei Y. G. Yang H. X. Zhu C. H. Deng J. J. Fan D. D. (2020). Hypoglycemic effect of ginsenoside Rg5 mediated partly by modulating gut microbiota dysbiosis in diabetic db/db mice. J. Agric. Food. Chem. 68, 5107–5117. 10.1021/acs.jafc.0c00605 32307991

[B24] Zhang L. H. Jia Y. L. Lin X. X. Zhang H. Q. Dong X. W. Zhao J. M. (2013). AD-1, a novel ginsenoside derivative, shows anti-lung cancer activity via activation of p38 MAPK pathway and generation of reactive oxygen species. Biochim. Biophys. Acta 1830, 4148–4159. 10.1016/j.bbagen.2013.04.008 (23583729

[B25] Zhao C. Su G. Y. Wang X. D. Zhang X. S. Guo S. Zhao Y. Q. (2016). Antitumor activity of ginseng sapogenins, 25-OH-PPD and 25-OCH3-PPD, on gastric cancer cells. Biotechnol. Lett. 38, 43–50. 10.1007/s10529-015-1964-4 26428367

[B26] Zhu S. Jiao W. h. Xu Y. L. Hou L. J. Li H. Shao J. R. (2021). Palmitic acid inhibits prostate cancer cell proliferation and metastasis by suppressing the PI3K/Akt pathway. Life. Sci. 286, 120046. 10.1016/j.lfs.2021.120046 34653428

