# Supplementary figures and images for: Integrating network pharmacology and experimental validation to explore the effect and mechanism of AD-1 in the treatment of colorectal cancer
Source: Front Pharmacol. 2023 May 22;14:1159712. doi: 10.3389/fphar.2023.1159712 (PMC10239872; doi:10.3389/fphar.2023.1159712)

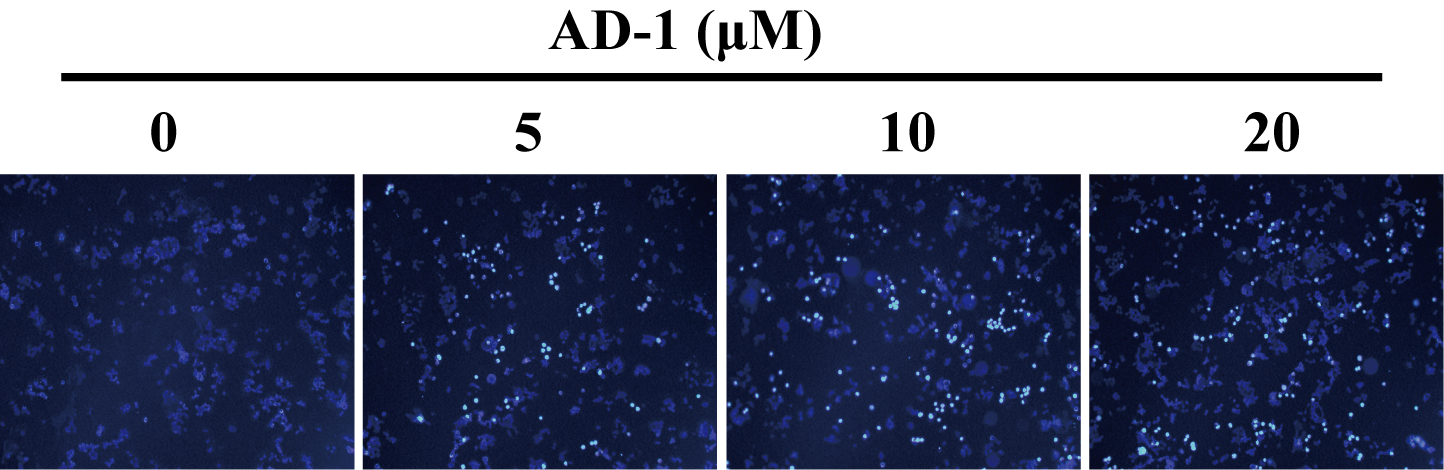

Supplement: Supplementary file 1 [file Image1.TIF]
